# Case of Death from Oxymuriate of Mercury

**Published:** 1819-03

**Authors:** 


*For the London Medical and Physical Journal.*

### *Case of Death from Oxymuriate of Mercury*; by ADJUTOR.

THE frequent occurrence of suicide from corrosive poisons, and our imperfect knowledge of certain antidotes for them, makes every case, whether of fortunate termination or not, a subject of considerable importance, not only in a pathological, but also in a juridical, point of view. Should the relation of the following case be considered worthy a place in your Journal, I shall consider myself honoured by its insertion.

In company with a friend I had the pleasure to assist, I visited, on January 22 of this year, at 11 o'clock P.M. a servant-girl, of short stature and robust habit, who, in consequence of some disappointment in love, formed the determination of destroying herself; and, after eating a hearty supper of bread and cheese, with cold bacon, and a quantity of malt liquor, dissolved about a drachm of hydrargyri oxymurias, and immediately swallowed it. A few minutes had scarcely elapsed, before the family were alarmed by the groans of the girl, whom they found on her knees complaining of a most severe burning pain at the stomach, extending upwards into the throat and mouth. This was soon followed by violent vomiting of what she had taken for supper, mixed with large quantities of viscid mucus, which before our arrival had filled a good-sized chamber-pot. Half a drachm of zinci sulphas was immediately given, and succeeded, from time to time, by warm water beat up with whites of eggs, gruel, &c. The emetic was repeated in an hour after. Upon the exhibition of the albuminous liquor, the evacuations put on that flaky, curd-like appearance spoken of in M. Orfila's system of Toxicology.

At 3 A.M. the vomitings were of a bilious kind, mixed with blood. She had also about this time three stools, of a dark and highly-offensive kind. Pulse 100, small, and tight. After this the pain abated, and she dozed at times, but was aroused by a recurrence of the pain. Countenance expressive of the greatest anxiety. At 9 A.M. I left her apparently better. As her bowels had not been opened since three o'clock, an oleaginous aperient mixture was ordered, with fomentations to the epigastrium, and a continuance of gruel, white of eggs, &c. Saw her again in the evening: pain in the stomach easier, but complains of the throat, which is greatly inflamed. A cooling gargle was ordered for her, and glysters to be given every two hours.

23d, 3 P.M.—Bowels have not yet been acted upon, nor has she made any water since yesterday morning; no tension of the abdomen, neither does pressure produce pain. The catheter was with some difficulty introduced, in consequence of the inflammation and thickening of the urethra and bladder: a few drops of urine only escaped. Saline aperients, with diuretics, were ordered, and the glysters to be continued.

24th.—The bowels have been acted upon, but no water secreted upon again using the catheter. Stomach easier; but the throat continues inflamed, and with a sense of constriction, so as to prevent swallowing without considerable efforts. The system slightly affected with the absorbed hydrargyrus; teeth painful, and rather loose; saliva rather increased in quantity. To have beef-tea and other mucilaginous drinks frequently.

25th, 9 o'clock A.M.—Found her sitting by the fire, and, in answer to my enquiries, says she is better; but, upon more attentive examination, evidently sinking. Has had frequent stools since yesterday, of a dark and highly-offensive kind. System more affected; teeth very loose, and a large discharge of saliva; breath exceedingly fetid; very little pain on pressure of the abdomen, which is not sensibly tumefied. Pulse scarcely to be felt; and countenance indicative of great debility. Catheter introduced without any water being discharged, and returned of a dark-blue colour, which required considerable friction with chalk to remove.

From this time she gradually became more exhausted, and, without experiencing a return of the pain, died on Monday at 3 P.M. about ninety hours after the ingestion of the poison.

I regret that business of a particular kind prevented the examination of the body till early on Wednesday morning, when the putrid state of it presented too many obstacles to be easily surmounted. Upon inspecting it, the most offensive odour exhaled from it I ever experienced. The abdomen was greatly distended, and of a very dark colour. The face was much distorted, and the mouth filled with viscid phlegm.

This case demonstrates, that, notwithstanding the excellent account given by M. Orfila of the action of albumina on the hydrarg. oxymurias, we are not to consider it as possessing the power of completely decomposing it, so as to form an inert preparation of that metal; and consequently it becomes every practitioner, when called to a patient having taken it, to expedite its expulsion by every means most likely to do so. Albumine should, however, on no account be left unused; the effect it had in this case of allaying pain was certainly great.

How far this salt may be given in patients labouring under diabetes, I have not had an opportunity of trying. In some cases of poisoning from hydrargyri oxymurias, related by Mr. Valentine, in the Edinburgh Medical and Surgical Journal, the effects produced upon the urinary secretion and bladder accord precisely with what I have now given.

*Dec.* 19, 1818.

